# The InterSECT Framework: a proposed model for explaining population-level trends in substance use and emotional concerns

**DOI:** 10.1093/aje/kwae013

**Published:** 2024-02-22

**Authors:** Jillian Halladay, Matthew Sunderland, Cath Chapman, Maree Teesson, Tim Slade

**Affiliations:** The Matilda Centre for Research in Mental Health and Substance Use, University of Sydney, Sydney 2006, Camperdown 2006, New South Wales, Australia; School of Nursing, McMaster University, Hamilton, Ontario L8S 4K1, Canada; Peter Boris Centre for Addictions Research, McMaster University / St. Joseph's Healthcare Hamilton, Hamilton, Ontario L8P 3P2, Canada; The Matilda Centre for Research in Mental Health and Substance Use, University of Sydney, Sydney 2006, Camperdown 2006, New South Wales, Australia; The Matilda Centre for Research in Mental Health and Substance Use, University of Sydney, Sydney 2006, Camperdown 2006, New South Wales, Australia; The Matilda Centre for Research in Mental Health and Substance Use, University of Sydney, Sydney 2006, Camperdown 2006, New South Wales, Australia; The Matilda Centre for Research in Mental Health and Substance Use, University of Sydney, Sydney 2006, Camperdown 2006, New South Wales, Australia

**Keywords:** alcohol, cannabis, tobacco, depression, anxiety, adolescence, trends

## Abstract

Across high-income countries, adolescent emotional concerns have been increasing in prevalence over the past two decades and it is unclear why this is occurring, including whether and how substance use relates to these changing trends. On the other hand, substance use has been generally declining, and little is known about the role of emotional concerns in these trends. Several studies have explored the changes in co-occurring substance use and emotional concerns among adolescents over time, with mixed results and inconsistent messaging about the implications of the findings. In response, we developed a theoretical framework for exploring the intersection between trends in substance use and emotional concerns (InterSECT Framework). This framework includes a discussion and related examples for 3 core hypotheses: (1) strengthening of co-occurrence, or the “hardening” hypothesis; (2) co-occurrence staying the same, or the “consistency” hypothesis; and (3) weakening of co-occurrence, or the “decoupling” hypothesis. This framework seeks to guide the conceptualization, evaluation, and understanding of changes in the co-occurrence of substance use and emotional concerns over time, including outlining a research agenda informed by pre-existing research and youth perspectives.

Over the past two decades, there have been two seemingly conflicting epidemiologic trends observed among adolescents across high-income countries. The first—and more concerning—trend is the striking escalation in the prevalence of mental ill health among adolescents. Namely, increases have been observed in rates of emotional concerns related to depression, anxiety, suicidality, and psychological distress, with some estimates suggesting a doubling over the past decade.^1–6^ These general population trends are mirrored by increases in emotional and self-harm–related presentations to the emergency department and subsequent psychiatric hospitalizations among adolescents.^7^^,^^8^ The second trend is the reduction, plateauing, or only modest increases in rates of common substance use among adolescents. There have been large reductions in adolescent cigarette smoking,^9^ moderate-large reductions in alcohol use,^10^ and slight reductions in cannabis use,^11^ although these trends have been plateauing in recent years, with some evidence of possible increases in cannabis use.^12^^,^^13^ Given the high prevalence and negative impact of co-occurring substance use and emotional concerns,^14–17^ it is unclear why these seemingly interconnected adolescent concerns are showing such different population-level trends. There has been a global effort to dismantle siloed systems of mental health and substance use treatment due to common comorbidity,^18^ although less attention has been paid to understanding co-occurrence from a population, preventive perspective. For example, few universal school prevention trials exist globally that target both substance use and emotional concerns.^19–21^ With a desire to halt or reverse the upward escalation in emotional concerns, and to maintain the downward trends in substance use, understanding how these concerns are operating together over time is important to inform effective, contemporary prevention and early intervention efforts.

The reasons for co-occurring substance use and emotional concerns are still in question, with most theories pointing towards multiple, diverse, bidirectional, and multilevel (eg, biological, psychological, social, contextual) pathways to co-occurrence.^1^^,^^22–24^ It is important to recognize that although co-occurrence is common, it is not a universal experience. According to an adapted 4-quadrant model, there may be youth who report low substance use and emotional concerns (quadrant 1), low substance use but high emotional concerns (quadrant 2), high substance use but low emotional concerns (quadrant 3), and high concerns in both (quadrant 4)^25^^,^^26^ (Figure 1). The diverging independent population trends add a layer of curiosity that suggests that the prevalence of youth falling into these different quadrants and the related risk and protective contexts may be changing. For example, the overall proportion of youth in quadrants 3 and 4 would have increased with escalations in emotional concerns, while the overall proportion of youth in quadrants 2 and 4 would have decreased alongside the reductions in substance use. However, we do not yet know whether changes are similar across youth with and without co-occurring concerns (eg, quadrant 4 vs. 2 and 3). We need a clearer understanding of how substance use has had and continues to have impacts on trends in emotional concerns and vice versa. Are youth who use substances experiencing different changes in emotional concerns compared with youth who do not use substances? Are youth with emotional concerns experiencing different changes in substance use compared with youth who do not report emotional concerns? This gap in knowledge prevents us from understanding the risk and protective contextual factors driving change, which limits forecasting of future trends and inhibits efficient and effective population policy and prevention.

**Figure 1:**
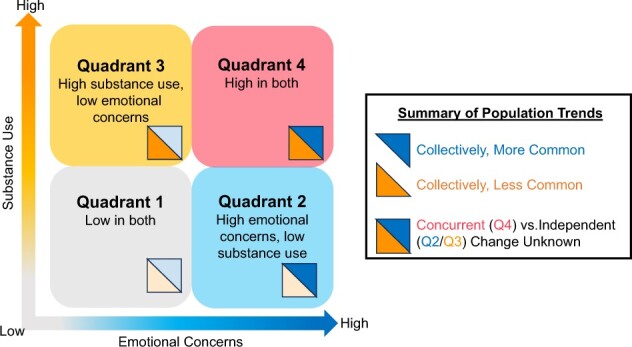
Adapted 4-quadrant model of co-occurring substance use and emotional concerns.

Several studies have explored the changes in co-occurring substance use and emotional concerns among adolescents over time, with mixed results depending on the specific type and measurement of substance use, the type and measurement of emotional concerns (75% of studies explore depressive symptoms), the years observed, and the sample characteristics.^27–38^ Further, there is inconsistent messaging about the implications of the findings. In response, we developed a theoretical framework to guide the conceptualization, evaluation, and understanding of changes in the co-occurrence of substance use and emotional concerns over time, including outlining a research agenda informed by preexisting research and youth perspectives.

## Youth consultation

Guided by the notion of “nothing about us without us”^39^^,^^40^ and “shifting from designing *for* users to designing *with* users,”^41^ an Australian National Health and Medical Research Council–funded Youth Advisory Board (YAB) was consulted during the development of this framework (see Prior et al^42^ for detailed information). YAB members were selected through a competitive national recruitment process prioritizing including at least 1 member from each state/territory in Australia, at least 1 Aboriginal or Torres Strait Islander person, and at least 1 person identifying as LGBTQIA+. The YAB consulted for this work was made up of - linguistically, culturally, sexuality, and gender diverse youth 16-25 years of age from metropolitan, rural, and regional areas across Australia, with an interest in substance use and/or emotional concerns via their own lived experience or that of their families and/or communities. During an annual in-person YAB meeting (youth compensated $100 [Australian] per day + travel costs), a detailed YAB review was conducted to co-produce hypothetical mechanisms related to trends.^42^ This included an audio-recorded 60-minute face-to-face extended group discussion facilitated by Google (Mountain View, CA) Jamboard (an interactive whiteboard for collective brainstorming) focused on 4 scaffolded questions (paraphrased, see Appendix S1 for the full consultation guide): (1) Do you have any ideas about why this [increases in youth emotion concerns] might be happening? (2) Do you have any ideas about why this [decreases in youth substance use] might be happening? (3) If we assume that emotional concerns became more common among young people using substances over time, what might be the reasons [and vice-versa]? And (4) is there anything else you think researchers or policymakers looking into these trends should know or consider? YAB members were provided with a brief prior to the session and a summary following the consultation. Youth perspectives on mechanisms of change were derived from synthesizing and identifying themes across Jamboard responses, minutes taken by a YAB coordinator, and direct quotes obtained through transcribed audio recordings.

## The InterSECT Framework: a theoretical framework for exploring the intersection of trends in substance use and emotional concerns

**Figure 2:**
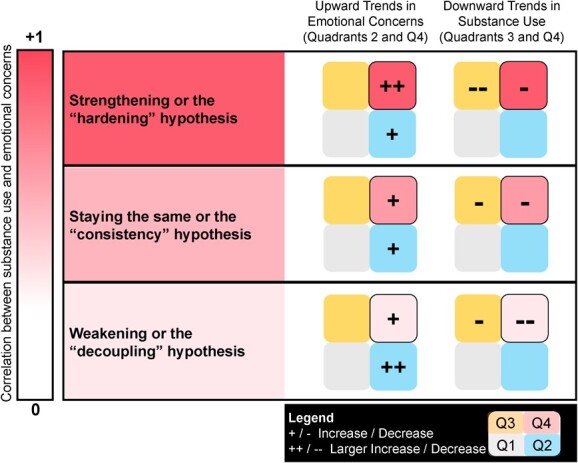
The InterSECT Framework: A continuum of trends in co-occurring substance use and emotional concerns. The continuum is interpreted relative to a start point assumed to be some level of co-occurrence or positive association.

Thinking of the full spectrum of possibilities related to co-occurrence: One end is anchored with substance use and emotional concerns always co-occurring (completely positively coupled) while the other end is anchored with substance use and emotional concerns never co-occurring (completely negatively coupled). We assume these extremes are unrealistic, with the truth likely somewhere in between no correlation (*r* = 0) and a perfect positive correlation (*r* = +1). The specific location on this spectrum suggests different shifts in the balance of shared and independent risk and protective factors and contexts over time. See Figure 2 for the InterSECT Framework.

It is unlikely that a single factor is responsible for change over time, but rather a collection of factors is operating at multiple levels, including individual (eg, pubertal timing, sex, genetics), interpersonal (eg, parental and peer relationships), and contextual (eg, societal, cultural, economic, political factors, and events). Given that the InterSECT Framework was driven largely by growing research in population trends—and thus between-person study designs—we focus the present discussion on risk and protective contexts*,* which may be driven by generational (ie, birth cohort) or period (ie, year of data collection) changes in the presence, meaning, and impact of risk and protective factors within the population over time. While independent contextual factors may be contributing to changing trends in these phenomena separate from one another (eg, factors exclusively related to substance use trends that do not have an impact on emotional concerns and vice versa), we focus on the role of shared contextual factors that relate to both substance use and emotional concerns, and thus their co-occurrence. New shared risk or protective factors and/or existing risk or protective factors can strengthen, stay the same, and/or weaken in impact. There may be multiple factors changing over time in different ways including: (1) replacement effects, whereby a decrease in one risk/protective factor is replaced by another new or strengthening risk/protective factor; or (2) offsetting effects, whereby simultaneous increase in risks is offset by an increase in protection (and vice versa).

In the next section we summarize the existing literature with respect to changing trends in emotional concerns and substance use and propose hypothetical mechanisms explaining the changing trends. The hypothetical mechanisms are based on prior hypotheses published in the existing literature^27–38^ as well as consultations with the YAB. Where available, additional mechanistic research or expert commentaries were used to guide hypotheses (for examples, see the references list^6^^,^^21^^,^^36^^,^^43–61^). These are hypothetical mechanisms (not yet proven) that are not meant to represent an exhaustive list, but rather are presented to provide an evidence- and youth-informed launching point for future research investigations. Please see Table S1 (available at https://doi.org/10.1093/aje/kwae013) for further details and examples of data- and youth- driven mechanisms.

### Strengthening or the “hardening” hypothesis

#### The hypothesis

Where strengthening is observed, emotional concerns are increasing among adolescents using substances at a faster rate than adolescents not using substances (ie, quadrant 4 increasing, more so than quadrant 2), and thus adolescents using substances are more likely to report co-occurring emotional concerns than before. Considering the other direction of relationships, strengthening can also occur where declines in substance use are lower among adolescents with emotional concerns (ie, quadrant 4 decreasing, less so than quadrant 3), suggesting that adolescents with emotional concerns are more likely than those without to use substances than before. To date, 4 trend studies related to alcohol use,^28^^,^^31^^,^^32^^,^^38^ 2 related to smoking,^27^^,^^28^ and 2 related to cannabis^34^^,^^36^ have demonstrated strengthening (See Table 1). This suggests that the collective magnitude of shared contextual risk factors of co-occurrence has increased relative to the magnitude of shared contextual protective factors; this could be due to combinations of collective shared risks increasing and/or shared protection decreasing. On balance, relative to the prior characteristics of shared risk and protective contexts, the contemporary cumulative shared risks (new and existing) outweigh the cumulative shared protective factors within the population. Here, contextual mechanism(s) driving emotional concerns in the population upward are likely to be at least partially positively related to substance use (ie, shared risks), and/or mechanism(s) driving substance use downward are only (or more) effective for those without emotional concerns (ie, moderated by emotional concerns).

**Table 1:** Summary of existing research examining population trends in co-occurring substance use and emotional concerns among adolescents

**Reference No.**	**First author (year)**	**Location**	**Years (no. of waves)**	**Ages/grades (name of survey)**	**Substance use**	**Emotional concern**	**Analysis** ^**a**^
*Strengthening Hypothesis*							
28	Gage (2021)	United Kingdom	2005-2015 (2)	Ages 13-15 (Avon Longitudinal Study of Parents and Children and the Millennium Cohort Study)	Alcohol (lifetime); smoking (lifetime)	Depressive symptoms (Short Moods and Feelings Questionnaire)	S➔E
31	Ng Fat (2018)	England	2005-2015 (11)	Ages 16-24 (Health Survey for England)	Alcohol (lifetime non-drinking)	Mental health and wellbeing (General Health Questionnaire, Warwick-Edinburgh Mental Wellbeing Scale)	E➔S
32	Torikka (2017)	Finland	2000-2011 (6)	Grades 8-9, ages 14-16 (The School Health Promotion Study of the National Institute for Health and Welfare)	Alcohol (weekly drinking, weekly drunkenness)	Depressive symptoms (R-Beck Depression Inventory)	E➔S
38	Pape (2021)	Norway	2002-2012/2013 (2)	Ages 14-17 (Young in Norway 2002, Quality assurance system 2012-13)	Alcohol (past year any intoxication)	Depressive symptoms (Depressive Mood Inventory)	E➔S
27	Chung (2014)	South Korea	2005-2011 (2)	Grades 9-11 (Korean Youth Risk Behavior Web-based Survey)	Smoking (past month)	Depressive symptom (daily sadness/hopelessness)	E➔S
34	Weinberger (2020)	United States	2004-2016 (13)	Ages 12-17 (National Survey on Drug Use and Health)	Cannabis (past month)	Major Depressive Episode (DSM-IV criteria)	E➔S
36	Askari (2021)^b^	United States	1991-2018 (28)	Grades 8, 10, 12 (Monitoring the Future)	Cannabis (past month)	Internalizing symptoms (8 questions related to self-esteem and self-derogation)	S➔E
*Consistency Hypothesis*							
28	Gage (2021)	United Kingdom	2005-2015 (2)	Ages 13-15 (Avon Longitudinal Study of Parents and Children and the Millennium Cohort Study)	Alcohol (heavy); smoking (weekly); cannabis (lifetime)	Depressive symptoms (Short Moods and Feelings Questionnaire)	S➔E
29	Kahn (2022)	United States	2011-2017 (4)	Grades 9-12 (Youth Risk Behavior Survey)	Alcohol (past month any, binge drinking [≥5]); smoking (past month); cannabis (past month low frequency)	Suicidality (ideation, plans, attempts, attempts requiring medical attention)	S➔E
33	Weinberger (2017)	United States	2005-2013 (9)	Ages 12-17 (National Survey on Drug Use and Health)	Smoking (past month daily, nondaily)	Major depressive episode (DSM-IV criteria)	E➔S
36	Askari (2021)^b^	United States	1991-2018 (28)	Grades 8, 10, 12 (Monitoring the Future)	Smoking (past month)	Internalizing symptoms (8 questions related to self-esteem and self-derogation)	S➔E
38	Pape (2021)	Norway	2002-2012/13 (2)	Ages 14-17 (Young in Norway 2002, Quality assurance system 2012-13)	Alcohol (past year frequent 6+ intoxication); cannabis use (past year)	Depressive symptoms (Depressive Mood Inventory)	E➔S
35	Lu (2021)	United States	2011-2019 (9)	Ages 12-17 (National Survey on Drug Use and Health)	SUD (past year DSM-IV criteria)	Major Depressive Episode (past year DSM-IV criteria)	➔ S/E
37	Mojtabai (2016)	United States	2005-2014 (10)	Ages 12-17 (National Survey on Drug Use and Health)	SUD (past year DSM-IV criteria)	Major Depressive Episode (past year DSM-IV criteria)	S➔E
*Weakening Hypothesis*							
30	Keyes (2020)	United States	1991-2018 (28)	Grade 12 (Monitoring the Future)	Alcohol (past 2-week binge drinking)	Depressive symptoms (4 items)	E➔S
36	Askari (2021)^b^	United States	1991-2018 (28)	Grades 8, 10, 12 (Monitoring the Future)	Alcohol (past 2-week binge drinking)	Internalizing symptoms (8 questions related to self-esteem and self-derogation)	S➔E
27	Chung (2014)	United States	2005-2011 (2)	Grades 9-11 (Youth Risk Behavior Survey)	Smoking (past month)	Depressive symptom (daily sadness/hopelessness)	E➔S
29	Kahn (2022)	United States	2011-2017 (4)	Grades 9-12 (Youth Risk Behavior Survey)	Cannabis (past month frequent [≥20 times])	Suicidality (ideation, plans, attempt, attempt requiring medical attention)	S➔E

Abbreviations: DSM, *Diagnostic and Statistical Manual*, *Fourth Edition*; SUD, substance use disorder.

^a^ S➔E: substance use as the independent and emotional concerns as the dependent variables; E➔S: emotional concerns as the independent and substance use as the dependent variables; ➔S/E: comorbid substance use and emotional concerns as the dependent variable.

^b^ Analyzed temporal changes by decade and also examined nuanced patterns with co-occurring externalizing symptoms. The effects related to internalizing symptoms only are reported in this table.

#### Evidence- and youth-informed perspectives

Contextual shifts may strengthen the co-occurrence of substance use and emotional concerns through changes in public perceptions, access to alternative reinforcers, peer pressure, and biochemical properties. Increased knowledge of the harms associated with substance use, whether through public health messaging or education, may lead to denormalization of substance use, reduced acceptability, and greater shame and stigma around continued use.^44^ Individuals who continue to use despite changing societal pressures may have more underlying risk factors for both mental health and substance use concerns, such as a tendency for risk-taking or susceptibility to negative emotions. Conversely, some substances may be increasingly perceived to have therapeutic benefits (eg, cannabis), which could contribute to increased self-medication among adolescents with emotional concerns.^46^ In addition, increasing financial and occupational insecurity may limit access to substance-free alternative activities and coping strategies (eg, employment, entertainment, health care, mental health supports, structured peer activities).^47^^,^^48^ The impact of limited alternatives may be amplified by difficulties tolerating boredom and loneliness, possibly driven by immediate gratification and reliance on social media. With fewer in-person peer interactions (due to greater reliance on social media and/or heightened parental monitoring), developing meaningful friendships may be challenging.^6^^,^^49^ Given that peer pressure still exists and substance use remains a status symbol in certain social circles, for some, the desire to belong to any peer group may outweigh the perceived risks of belonging to the “wrong crowd.” This may be particularly pronounced for those in remote or rural areas, or in socioeconomically disadvantaged families. Finally, increased potency of some substances, such as cannabis, and interactions between substances and new psychotropic medications may strengthen the biological causal pathways between use and emotional concerns.^45^

### Staying the same or the “consistency” hypothesis

#### The hypothesis

Where the magnitude of the association between substance use and emotional concerns stays the same over time in the population, the increases in emotional concerns are similar for adolescents who use or do not use substances (ie, similar increases in quadrant 4 and quadrant 2), and/or the decreases in substance use are similar for adolescents with and without emotional concerns (ie, similar decreases in quadrant 4 and quadrant 3). To date, 2 trend studies related to alcohol use,^28^^,^^29^ 4 related to smoking,^28^^,^^29^^,^^33^^,^^36^ 3 related to cannabis,^28^^,^^29^^,^^38^ and 2 related to substance use disorders^35^^,^^37^ have shown consistency in associations over time (See Table 1). This suggests that the magnitude and balance of shared risk and protective contextual factors have remained consistent*,* although the combinations and mechanisms of factors may be changing; there may be consistency in the nature of the existing risk and protective context, replacement of weakened risks with new or strengthened risks, replacement of protective factors, and/or offsetting of changes in risks with equal changes in protection. Here, the contextual mechanism(s) driving distress upward are likely unique to emotional concerns (ie, independent risks), and/or cumulative shared protective factors have strengthened to offset any increased shared risk. On the other hand, mechanism(s) driving substance use downward in this situation are likely unique to substance use (ie, independent protective factors), and/or cumulative shared risk factors have strengthened to offset any increased shared protective factors.

#### Evidence- and youth-informed perspectives

Adolescents today face a variety of contextual shifts that may increase emotional concerns, regardless of whether or not they use substances. These shifts include new unrealistic expectations in work and lifestyle, increasingly anxiogenic environments, shifts in how youth spend their time and connect, and decreasing human connection. One major concern is the perception of “hustle culture” at work or school, which can lead to working outside of work hours without compensation and feeling unable to take a day off.^43^ In addition, social media exposes youth to unrealistic lifestyle and beauty standards, leading to upward comparisons and feelings of inadequacy.^50^ Alongside these pressures, adolescents are confronting greater uncertainty regarding climate change, political climate, and financial stability in their current and future lives.^47^^,^^51^ As a result, adolescents report feeling “time-poor” and struggle to find time outside of work or school to engage in leisure, self-care, or social activities. Additionally, today’s adolescents are less active, more sedentary, have poorer diets, sleep less, and feel lonelier.^52^^,^^53^ Decreased human connections may be due to focus on work/school, increased parental monitoring, and social media reducing the quality and depth of social connections.^6^^,^^54^ Social media also contributes to a heightened awareness and exposure to negative news and extreme polarized views on social and political issues. Of note, although the YAB members believe social media has amplified existing issues, they do not believe it is the root cause or the solution. On the other hand, substance-related policy changes (eg, changes in availability), introduction of prevention programs, or shifts in parental monitoring may be contributing to declines in substance use regardless of the presence or absence of co-occurring emotional concerns.^49^^,^^60^^,^^61^

### Weakening or the “decoupling” hypothesis

#### The hypothesis

Where the co-occurrence of emotional concerns and substance use is decreasing over time in the population, emotional concerns are increasing more so among adolescents who do not use substances in comparison with those that do (ie, quadrant 2 increasing, more so than quadrant 4), and/or substance use is decreasing, more so among those with emotional concerns (ie, quadrant 4 decreasing, more so than quadrant 3). To date, 2 trend studies related to alcohol use,^30^^,^^36^ 1 related to smoking,^27^ and 1 related to cannabis^29^ have shown weakening of associations between substance use and emotional concerns over time (See Table 1). The 2 studies related to alcohol demonstrated a complete decoupling, whereby binge drinking and emotional concerns were no longer related in recent years, whereas the other 2 studies still showed elevations in emotional concerns among adolescents who used substances (though weaker than years prior). This suggests that the magnitude of shared contextual risk factors of co-occurrence has decreased in relation to the magnitude of shared contextual protective factors, which may have increased due to combinations of collective risks weakening and/or protective factors strengthening. On balance, relative to the prior characteristics of shared risk and protective contexts, the contemporary cumulative protective factors (new and existing) outweigh the cumulative risk factors. Here, the key contextual mechanisms driving emotional concerns upward in the population are likely not positively related to substance use (ie, independent risks) or possibly inversely related with substance use (ie, risks for emotional concerns are protective for substance use) while mechanisms driving substance use downward are, at least partially, also related to lower emotional concerns (ie, shared protective factors).

#### Evidence- and youth-informed perspectives

Various contextual factors may be contributing to a slower increase in emotional concerns among those who use substances, and vice versa, such as changes in coping strategies, public perceptions and education, biological impacts, and externalizing pathways. With increasing discussions and education related to mental health and coping strategies,^55^^,^^56^ adolescents may be more likely to turn to nonsubstance coping strategies than they were in the past (eg, mindfulness, exercise, prescribed medication, therapy, peer support, social media). At the same time, more education and awareness of the harms of substance use and how to respond to peer pressure, alongside denormalization of substance use, may contribute to lower initiation and less pressure to use.^21^^,^^44^ Another possible factor is the changing biochemical properties of substances, which may result in possible antidepressant or anxiolytic effects of recreational drugs, particularly related to cannabis (although under investigation, this is not currently supported^57^). The later age of initiation of substance use may be resulting in fewer negative neurobiological and social implications of substance use later in adolescence and young adulthood.^44^^,^^58^ Furthermore, declines in co-occurrence may be explained by declines in risky behaviors more generally.^36^^,^^59^ There may also be factors that protect against substance use (eg, parental monitoring^49^) that are concurrently contributing to risk for emotional concerns (eg, fewer in-person peer interactions^6^^,^^49^).

## Mitigating methodological biases in future research

There are several methodological issues to pay close attention to when trying to understand population trends, and that may be leading to biased and incorrect conclusions, related to missingness, operationalization of variables, nonlinearity, and identification of mechanisms. First, there have been decreases in response rates and increases in missingness in surveys over time,^62^ making reporting and exploring missingness, adjusting for demographics, and leveraging contemporary missing data strategies increasingly important. To note, true change in the demographic makeup may itself be a direct mechanism of change (eg, decreasing substance use related to more immigrant youth from places with abstinence-based cultures). Second, the operationalization of substance use should be considered carefully in relation to type and patterns of use. All substances (alcohol, cannabis, smoking) may operate differently over time, although the use of multiple substances is common, and patterns of use may have different associations^25^; therefore, researchers should consider both trends in individual substances and patterns of multiple use (eg, number of substances, latent profiles). Third, emotional concerns should also be considered with respect to distinct types (depression, anxiety, distress, suicidality) and patterns of comorbidity using valid multiple-item or diagnostic measures. Given that some theorize that the increase in emotional concerns may not actually be reflective of true increases but rather willingness to report,^63^ establishing measurement invariance across time is critical.^64^

It is also possible that trends in the co-occurrence are nonlinear, related to birth cohorts rather than survey year, and are driven by unmeasured mechanisms. Where possible, researchers should explore nonlinear models (eg, polynomials or time-varying effect modeling^65^) and models that disentangle age, period, and cohort effects (eg, Bell^66^). Further, although existing repeated cross-sectional population surveys can characterize the direction and magnitude of trends, and provide insight into contextual changes, they may not be able to provide answers to what is driving between-person changes over time. For example, certain variables may not be measured at all (eg, climate anxiety) or may not be measured with enough specificity (eg, social media, time use) to be able to explore mechanisms of between-person change across time. Thus, a qualitative sociological perspective may be needed to understand historical mechanisms, and ongoing surveys may need to be amended to include new or more nuanced variables moving forward (eg, Caluzzi et al^44^).

Last, repeated cross-sectional population surveys cannot infer within-person change and thus longitudinal cohort studies are required for understanding within-person causal pathways.^67^ Population-level data gives us answers to whether the pattern of adolescents reporting higher levels of substance use (compared to those with none) or a specific type of use (eg, prior-month binge drinking) correlates with higher emotional concerns or a specific threshold of emotional concerns (eg, meeting criteria for depression). This approach can be particularly helpful for determining whether substance use co-occurs with emotional concerns at the same rate over time (and whether use is more or less of a “flag” for emotional concerns today than in the past and vice versa) and for exploring period- or cohort-related contextual mechanisms and interventions. Conversely, individual-level longitudinal data provides answers to the more nuanced questions of whether adolescents using substances more or less than their peers on average (grand-mean centered) and/or more or less than their personal norms (person-mean centered) correlates with, leads to, or is a consequence of changes in their emotional concerns. This enables explorations of within-person mechanisms, which can inform any individual-focused interventions or education.

## Discussion

Across high-income countries, adolescent emotional concerns have been increasing in prevalence over the past two decades while substance use has generally been declining, and it is unclear why this is occurring, including whether and how these trends relate to one another. First, we need to replicate trends related to co-occurrence across samples, measures, and countries. As voiced by youth, this exploration needs to include understanding whether these trends are having a disproportionate impact on certain subgroups of youth (eg, based on socioeconomic status, geographical location, those with a family history of substance use or mental health problems, or those with experiences of trauma). Second, we need to identify and explore possible mechanisms related to these trends, which may require the application of interdisciplinary approaches (eg, macroeconomics, sociology, systems-based frameworks) and a focus on both between- and within-person designs. Third, we cannot assume that prior risk and protective factors are operating similarly today, and thus we also need to reestimate associations and related factors regarding whether and how co-occurrence presents and operates today (rather than relying on historical studies).

Possible mechanisms for future exploration include multilevel (individual, contextual, societal) shifts in: (1) how youth spend their time and connect, including movement behaviors, parental monitoring, leisure, social media, and loneliness/belongingness (eg, Kreski^54^); (2) attitudes, perceptions, and motives, such as perceived risk of harm, social acceptability, stigma and shame, peer pressure, and coping motives (eg, Caluzzi et al^44^); (3) academic- and work-related pressures, expectations, and cultures (eg, Burgess et al^43^); (4) anxiogenic, uncertain environments, such as in relation to economic, political, and climate crises (eg, Grant et al^51^); (5) biological impacts of substance use, including increasing potency of cannabis, interactions with psychotropic medications, or later age of onset (eg, Freeman et al^45^); and (6) general risky behaviors that may possibly suggest a unitary downward trend in externalizing traits, cascade of declines in related risky behaviors, or interactions between internalizing and externalizing concerns (eg, Askari et al^36^ and Grucza et al^59^). Youth voiced particular concerns about: (1) having too much to do with too little time, and inadequate financial compensation to meet demands of rising costs of living; and (2) decreasing time and ability to connect with others, develop close relationships, and feel a sense of belonging.

Youth are calling for more solution-focused prevention and early-intervention research and policies, focused on identifying and responding to the root mechanisms of these trends. Overall, the framework presented here can guide future researchers and policymakers in the design, interpretation, and actions relevant for future studies seeking to explore trends in co-occurrence over time.

## Supplementary Material

AJE-00347-2023_Supplementary_Data_Final_kwae013
