# Supplementary material for: The InterSECT Framework: a proposed model for explaining population-level trends in substance use and emotional concerns
Source: Am J Epidemiol. 2024 Feb 22;193(8):1066–74. doi: 10.1093/aje/kwae013 (PMC11299025; doi:10.1093/aje/kwae013)
Supplement: AJE-00347-2023_Supplementary_Data_Final_kwae013 [file aje-00347-2023_supplementary_data_final_kwae013.docx]

**The InterSECT framework: A proposed model for explaining population-level trends in substance use and emotional concerns**

Jillian Halladay, Matthew Sunderland, Cath Chapman, Maree Teesson, Tim Slade

**Appendix S1**: Youth Advisory Board Consultation Guide…………………………………2

**Table S1:** Youth Advisory Board Summary………………………………………………..4

**Appendix S1**: Youth Advisory Board Consultation Guide

**Question 1.** Emotional problems include things like depression symptoms (like feeling so sad nothing can cheer you up) or anxiety symptoms (like feeling overwhelming worry). These problems have been becoming **more common** among young people around the world over the past 10 years. Do you have any ideas about why this might be happening?

- **Probe**: What are the first things that come to mind?
  - **Prompt**: Any other individual, interpersonal, contextual, societal factors?
- **Probe**: Thinking of yourself, your friends, or your classmates/colleagues, what are the main things that cause emotional concerns?
  - **Prompt**: Some examples may be related to changes in how youth spend their time, changes in attitudes about mental health, changes in global and political climates.
  - **Prompt**: Some specific examples may be more social media use, less sleep and exercise, more climate anxiety.

[Open ended, ~15mins, Goal: inform quadrant 2 hypotheses and the consistency hypothesis]

**Question 2**. Substance use (like alcohol, cannabis, and cigarette use) has been becoming **less** **common** among young people around the world over the past 10 years. Do you have any ideas about why this might be happening?

- **Probe**: What are the first things that come to mind?
  - **Prompt**: Any other individual, interpersonal, contextual, societal factors?
- **Probe**: If you do not use substances (or if any of your friends do not use substances), what may be the main reasons why?
  - **Prompt**: Some examples may be related to changes in how youth spend their time, changes in attitudes about substances, or greater economic disadvantage .
  - **Prompt**: Some specific examples may be more monitoring from parents, less peer pressure to use substances, or more negative attitudes about substances like cigarette smoking.

[Open ended, ~15 mins, Goal: inform Quadrant 3 and the decoupling hypothesis]

**Question 3**. Even though substance use has gone down, some young people still use substances. Based on past research, young people who use substances are more likely to report emotional problems than those who do not use substances. What we don’t know is whether young people who use substances today experience more emotional concerns than young people who used substances 10 years ago. If we assume that emotional concerns **became more common** among young people using substances over time, what might be the reasons?

- **Probe**: What are the first things that come to mind?
- **Prompt**: Some examples may be higher potency of cannabis or more use of substances to cope with emotional problems today than in the past.
- **Probe**: The first question we asked today was about the general reasons why emotional concerns became more common over time.
  - Do any of the ideas we talked about in the first question apply here?
  - Are there things that may impact young people who use substances **more** than youth who do not use substances?
  - Are there any different reasons that are **specific** to young people who use substances?

[Open ended, ~20 mins, Goal: inform Quadrant 4 and primarily the strengthening hypothesis]

**Question 4**. Is there anything else you think researchers or policymakers looking into these trends should know or consider?

[Open ended, ~10 mins, Goal: inform future directions/considerations]

**Table S1: Youth Advisory Board Summary**

|  | **Hypothetical mechanisms** | **Examples or descriptions of mechanism** (informed by youth perspectives and prior research (1-33)) |
| --- | --- | --- |
| **Strengthening or “Hardening” Hypothesis** | | |
| Here, new risk factor(s) for distress are disproportionately impacting those who use substances and/or existing protective factors are weakening (though the latter is less likely). | | |
| **NEW OR STRENGTHENED RISK FACTORS**  (greater impacts among those who do use substances) | Increasing **shame** related to substance use* | Due to increasing education and messaging around harms of substance use, and decreasing public acceptance, there may be greater levels of shame associated with continued use of substances. This also suggests that those who continue to use despite changing societal pressures and perspectives may have more underlying risk factors related to both mental health and substance use concerns and less access to alternatives and supports (e.g., personality, susceptibility to negative emotions, socioeconomic deprivation, trauma). |
|  | Worsening **time** pressures | Using substances takes time (especially if using frequently) and thus may magnify perceived and actual time-deprivation. |
|  | Surviving **“Hustle Culture”** | Some adolescents who feel greater pressure to perform may use substances to stay awake or be more productive (see below for more information on hustle culture). |
|  | Desire for any type of **friendship** or connection with others* | Due to decreasing social connections (see below for more information of decreased connection), adolescents may use substances to fit in with family or peers who use substances. The desire for belonging to any group outweighs perceptions of the “wrong crowd.” Peer pressure still exists, and in some communities, may remain acceptable and a symbol of social status.  In particular, social media may lower social skills and reduce deep connections with others resulting in a greater perceived need to use substances to socialize (e.g., “social lubricant” effects of alcohol, or “calming” effects of cannabis), greater likelihood of giving into peer pressure (i.e., conformity motives), or using substance to fit in with *any* group even if the “wrong group.” |
|  | Increasing **financial stress and instability** | *Economic crises* may be disproportionately impacting adolescents from already disadvantaged populations who are at risk for both substance use and mental health concerns. |
|  | New **biochemical** **properties** of evolving substances or interactions with new psychotherapeutic medications | *New biochemical propert*ies of evolving substances (e.g., THC potency of cannabis) or *interactions* with new psychotherapeutic medications (e.g., nicotine and stimulants) may be increasing co-occurrence. |
|  | Increasing use of substances to **cope** with emotional concerns | - Escaping what is happening at home, trauma, or general awareness of negativity around the world.* - Not all adolescents have equal access to supports or education to learn alternative coping strategies. - Perceived therapeutic effects: For example, increases in perceived therapeutic benefits of cannabis use for anxiety and depression. Among those with ADHD, there may also be the use of nicotine for cognitive or attention effects or cannabis for perceived calming effects to offset symptoms. |
|  | Lack of access to **alternative activities*** | Adolescents may use substances to cope with boredom - which may be particularly pronounced among youth in remote/rural areas or in socioeconomically disadvantages families - due to lack of access to job opportunities, substance-free entertainment, healthcare, or coping strategies for dealing with boredom.   - Relatedly, *delay discounting* (transdiagnostic behavioural economic risk factor) may have increased due to a general shift towards access to and need for immediate rewards and gratification (e.g., phones, social media, email/internet). |
|  | Greater **risk taking** required to continue to use substances | Increased public awareness of harm (namely related to tobacco), restricted access, and lower acceptability, may result in adolescents who continue to use substances needing higher latent deviance/risk-taking traits than those who do not. |
| **Staying the Same or “Consistency” Hypothesis** | | |
| Here, new distinct drivers of distress are equally impacting those with and without substance use. Shared risk and protective factors may be changing but the overall balance remains the same. | | |
| **NEW OR STRENGTHENED RISK FACTORS**  (impacting all adolescents) | Increasing **unrealistic expectations*** | Two core types and mechanisms of unrealistic expectations:   - “Hustle culture” at work or school (i.e., working outside of work hours with little to no compensation, inability to take a day off). This is aligned with a shift toward a more *neoliberal* and *individualistic* society resulting in adolescents wanting to spend time more “productively” – increasing pressure to perform and be successful, with decreased perceived time for leisure and relaxation. - Social media increasing exposure to unrealistic lifestyle and beauty standards (i.e., exposure to “better” lives, upward comparison, youth feeling they cannot live up to what their body and lives “should” look like according to social media). |
|  | Increasing **financial stress** and instability* | Adolescents are increasingly worried about their economic present and futures due to housing crises, difficulty accessing affordable healthcare, rising costs of living, difficulty finding suitable work. |
|  | Increasing feelings of being “**time-poor**”* | Adolescents increasingly feel they do not have time outside of work or school (due to “hustle culture” and/or financial difficulties) to do other activities for leisure, self-care, or connecting with others. |
|  | Decreasing **human connection*** | Several mechanisms may be driving reduced human connections and increasing loneliness:   - Due to the increasing focus on and commitment to work, adolescents may feel as though there is less time to connect with others. - Technology and social media may have reduced the quality and depth of social connections due to technology (i.e., virtually connecting is lower quality and takes more time to establish deep connections, makes it difficult to understand context or emotion, removes physical touch [e.g., high fives, hugs], and reduces fun [i.e., not doing activities together]). - Increases in parental monitoring may decrease opportunities for in-person unstructured socialization with peers. |
|  | Decreased skills tolerating **boredom** or silence | Adolescents today constantly have access to people, tasks, and activities through technology (e.g., social media, multiple screens, games) with little opportunity to build skills to tolerate boredom or silence. |
|  | Increasing **climate uncertainty** | Increasing awareness and reporting of a possibly irreversible climate crisis with little political and global action to mitigate of reverse impacts makes the future of the world, food security, and disease uncertain for adolescents who still have many years of life ahead of them. |
|  | Changes in healthy **lifestyle behaviours** | Changes in movement related behaviours (e.g., worsening sleep, more sedentary time, less exercise) and in nutrition (e.g., increased processed foods). |
|  | Greater **negativity** and polarity in views | Due to technology and media, there is greater awareness and exposure to negative news and extreme polarized views on social and political issues (e.g., racism, discrimination, political views). Social media “pushes” this content negative on users. |
|  | Greater ability and **willingness** to talk about mental health | Adolescents may have greater awareness of what mental health and illness are, and thus be more willing and able to discuss and report symptoms. |
| **Weakening or “Decoupling” Hypothesis** | | |
| Here, new drivers of distress are disproportionately impact those who do not use substances (or concurrently driving down substance use). New (or strengthening of existing) shared protective factors may also be at play reducing co-occurring problems. | | |
|  | Increasing exposure to and use of **social media*** | While social media may be amplifying risks for emotional concerns, it may also be contributing to reductions in substance use due to:   - Exposure to medical professionals discussing harms of substance use. - Exposure to “influencers” (famous social media accounts) that often are based in entrepreneurship, healthy lifestyles (e.g., exercise, healthy eating), or recovery. Few influencers are known for, or famous, because of substance use. This communicates that substances are “not cool.” - More opportunities to socialize without substances and decreases in in-person socialization where substances may be present. - Heightened surveillance may decrease substance use for fear of negative social or occupational implications if posted online. |
| **NEW OR STRENGTHENED RISK FACTORS**  (greater impacts among those not using substances) | Increasing **parental monitoring** | While parental monitoring contributes to less unstructured, in-person socialization with peers which may contribute to increases in emotional concerns, this also may result in fewer opportunities to use substances. |
|  | **Too much to do, too little time to do it*** | Youth want to spend time more “productively” – increasing pressure to perform and be successful, with decreased perceived time for leisure and relaxation (including substance use). When youth do have time, want to spend time connecting with friends or family, engaging in self-care, or doing other leisure activities (“better things to do”). |
|  | Increasing **financial stress and instability** | Substances are expensive, and perhaps too expensive (youth want to spend money on other activities, like travel, or do not have enough money beyond daily living expenses). |
|  | More **risks** | - Increased awareness of legal trouble - Fear of contamination |
|  | **Body** awareness | Adolescents are increasingly worried about their health and body (including body image) and thus worries about how substances may negatively impact their health and attractiveness of their body. |
|  | Desire to break **generational cycles** | Greater ability to notice when family members behaviours are impacted by their substance use and wanting to avoid similar outcomes. There is also a heightened desire to break stereotypes. |
| **NEW OR STRENGTHENED PROTECTIVE FACTORS**  (impacting those who do use substances) | Changing **coping strategies** | Adolescents are turning to non-substance coping strategies (e.g., mindfulness, exercise, therapy, peer support, social media). |
|  | Shifting **public perceptions*** | Normalization, denormalization, stigma, and status changes decreases pressure to break rules or give in to peer pressure:   - Increased social and political *normalization* of substance use (namely, cannabis) may result in more adolescents trying or using substances without use being seen as “deviant” or “risky.” - *Denormalization* of substances (namely, alcohol and tobacco) or normalization of abstinence may decrease the presence and impact of peer pressure or need for adolescents to develop and employ refusal skills. - *Increase shame and stigma* related to substance use (namely tobacco), may prevent youth from initiating use. - Substance use *no longer a* *status symbol* or seen as *“cool.”* May make it more difficult to make friends if using substances. |
|  | Increasing awareness and **education** of harms* | - Greater awareness of side effects from school-based programs, greater translation of research evidence, social media health care providers. - More education on anticipating and responding to peer pressure. - Greater ability to notice when people’s behaviours are impacted by their substance use (e.g., seeing intoxicated people in public and able to link behaviours to substance use). - Multiple avenues of messaging that substance use is “bad” or harmful. |
|  | Changing **biochemical properties** of substance use | For example, certain strains of cannabis are being explored that may offer anti-depressant or anxiolytic effects. |
|  | Increasing access to targeted **early interventions** | Youth are more willing to seek help for mental health and substance use difficulties, and there may be greater access to targeted early interventions for co-occurring concerns (historically siloed systems are becoming more integrated). |
|  | Increasing **age of onset** | Shifting initiation of substance use later may be resulting in fewer negative neurobiological and social implications of substance use later in adolescence/young adulthood. |

* = most prominent in discussions with youth

**References**

1. Burgess A, Yeomans H, Fenton L. ‘More options… less time’in the ‘hustle culture’of ‘generation sensible’: Individualization and drinking decline among twenty‐first century young adults. *The British Journal of Sociology* 2022;73(4):903-18.

2. Caluzzi G, Livingston M, Holmes J, et al. Declining drinking among adolescents: are we seeing a denormalisation of drinking and a normalisation of non‐drinking? *Addiction* 2022;117(5):1204-12.

3. Freeman TP, Craft S, Wilson J, et al. Changes in delta‐9‐tetrahydrocannabinol (THC) and cannabidiol (CBD) concentrations in cannabis over time: systematic review and meta‐analysis. *Addiction* 2021;116(5):1000-10.

4. Pacek LR, Weinberger AH, Zhu J, et al. Rapid increase in the prevalence of cannabis use among people with depression in the United States, 2005–17: the role of differentially changing risk perceptions. *Addiction* 2020;115(5):935-43.

5. Frasquilho D, et al. Mental health outcomes in times of economic recession. *BMC Public Health* 2016;16(1).

6. Lechner CM, Tomasik MJ, Silbereisen RK. Preparing for uncertain careers: How youth deal with growing occupational uncertainties before the education-to-work transition. *Journal of Vocational Behavior* 2016;95:90-101.

7. Twenge JM, Joiner TE, Rogers ML, et al. Increases in depressive symptoms, suicide-related outcomes, and suicide rates among US adolescents after 2010 and links to increased new media screen time. *Clinical Psychological Science* 2018;6(1):3-17.

8. Vashishtha R, Livingston M, Pennay A, et al. Why is adolescent drinking declining? A systematic review and narrative synthesis. *Addiction Research & Theory* 2020;28(4):275-88.

9. Curran T, Hill AP. Perfectionism Is Increasing Over Time. *Psychological Bulletin* 2017.

10. Grant JB, Batterham PJ, Calear AL, et al. Recognising the anxiogenic environment as a driver of youth anxiety. *The Lancet Child & Adolescent Health* 2023.

11. Patalay P, Gage SH. Changes in millennial adolescent mental health and health-related behaviours over 10 years: A population cohort comparison study. *International Journal of Epidemiology* 2019;48(5):1650-64.

12. Madsen KR, Holstein BE, Damsgaard MT, et al. Trends in social inequality in loneliness among adolescents 1991–2014. *Journal of Public Health* 2018;41(2):e133-e40.

13. Kreski NT, Chen Q, Olfson M, et al. Time use and associations with internalizing symptoms from 1991 to 2019 among US adolescents. *SSM-population health* 2022;19:101181.

14. Seedaket S, Turnbull N, Phajan T, et al. Improving mental health literacy in adolescents: systematic review of supporting intervention studies. *Tropical Medicine & International Health* 2020;25(9):1055-64.

15. Dunning D, Tudor K, Radley L, et al. Do mindfulness-based programmes improve the cognitive skills, behaviour and mental health of children and adolescents? An updated meta-analysis of randomised controlled trials. *BMJ Ment Health* 2022;25(3):135-42.

16. Teesson M, Newton NC, Slade T, et al. Combined prevention for substance use, depression, and anxiety in adolescence: a cluster-randomised controlled trial of a digital online intervention. *The Lancet Digital Health* 2020;2(2):e74-e84.

17. Black N, Stockings E, Campbell G, et al. Cannabinoids for the treatment of mental disorders and symptoms of mental disorders: a systematic review and meta-analysis. *The Lancet Psychiatry* 2019;6(12):995-1010.

18. Livingston M, Callinan S, Vashishtha R, et al. Tracking the decline in Australian adolescent drinking into adulthood. *Addiction* 2022;117(5):1273-81.

19. Askari MS, Rutherford CG, Mauro PM, et al. Structure and trends of externalizing and internalizing psychiatric symptoms and gender differences among adolescents in the US from 1991 to 2018. *Social psychiatry and psychiatric epidemiology* 2021:1-12.

20. Grucza RA, Krueger RF, Agrawal A, et al. Declines in prevalence of adolescent substance use disorders and delinquent behaviors in the USA: a unitary trend? *Psychological medicine* 2018;48(9):1494-503.

21. Chung SS, Joung KH. Risk factors for current smoking among American and South Korean adolescents, 2005–2011. *Journal of Nursing Scholarship* 2014;46(6):408-15.

22. Gage SH, Patalay P. Associations between adolescent mental health and health-related behaviors in 2005 and 2015: A population cross-cohort study. *Journal of Adolescent Health* 2021;69(4):588-96.

23. Kahn GD, Wilcox HC. Marijuana use is associated with suicidal ideation and behavior among US adolescents at rates similar to tobacco and alcohol. *Archives of suicide research* 2022;26(2):520-33.

24. Keyes KM, Hamilton A, Patrick ME, et al. Diverging trends in the relationship between binge drinking and depressive symptoms among adolescents in the US from 1991 through 2018. *Journal of Adolescent Health* 2020;66(5):529-35.

25. Ng Fat L, Shelton N, Cable N. Investigating the growing trend of non-drinking among young people; analysis of repeated cross-sectional surveys in England 2005–2015. *BMC public health* 2018;18(1):1-10.

26. Torikka A, Kaltiala-Heino R, Luukkaala T, et al. Trends in alcohol use among adolescents from 2000 to 2011: the role of socioeconomic status and depression. *Alcohol and alcoholism* 2017;52(1):95-103.

27. Weinberger AH, Gbedemah M, Wall MM, et al. Depression among non-daily smokers compared to daily smokers and never-smokers in the United States: An emerging problem. *Nicotine & Tobacco Research* 2017;19(9):1062-72.

28. Weinberger AH, Zhu J, Lee J, et al. Cannabis use among youth in the United States, 2004–2016: Faster rate of increase among youth with depression. *Drug and alcohol dependence* 2020;209:107894.

29. Lu W, Muñoz-Laboy M, Sohler N, et al. Trends and disparities in treatment for co-occurring major depression and substance use disorders among US adolescents from 2011 to 2019. *JAMA Network Open* 2021;4(10):e2130280-e.

30. Mojtabai R, Olfson M, Han B. National trends in the prevalence and treatment of depression in adolescents and young adults. *Pediatrics* 2016;138(6).

31. Pape H, Rossow I. Less adolescent alcohol and cannabis use: More deviant user groups? *Drug and alcohol review* 2021;40(1):118-25.

32. Li S, Keogan S, Taylor K, et al. Decline of adolescent smoking in Ireland 1995–2015: trend analysis and associated factors. *BMJ open* 2018;8(4):e020708.

33. Miech R, Keyes KM, O'Malley PM, et al. The great decline in adolescent cigarette smoking since 2000: consequences for drug use among US adolescents. *Tobacco control* 2020;29(6):638-43.
